# Recovery and microbial host assignment of mobile genetic elements in complex microbiomes: insights from a spiked gut sample

**DOI:** 10.1128/msystems.01282-25

**Published:** 2026-01-26

**Authors:** Bram Bloemen, Maud Delvoye, Stefan Hoffman, Kathleen Marchal, Kevin Vanneste, Marie-Alice Fraiture, Nancy H. C. Roosens, Sigrid C. J. De Keersmaecker

**Affiliations:** 1 Transversal Activities in Applied Genomics, Sciensano 54513, Brussels, Belgium; 2 Department of Plant Biotechnology and Bioinformatics, Ghent University, Zwijnaarde, Belgium; 3 Department of Information Technology, Ghent University, Zwijnaarde, Belgium; Child Health Research Foundation, Dhaka, Bangladesh

**Keywords:** antimicrobial resistance, microbiome, nanopore sequencing, mobile genetic elements, MGE-host prediction, AMR

## Abstract

Mobile genetic elements (MGEs) are major drivers of horizontal gene transfer, including the spread of antimicrobial resistance (AMR) genes. However, determining the microbial host of an MGE in complex microbiomes remains challenging. Here, we spike a niche-aspecific *Bacillus velezensis* strain carrying a plasmid and linear phage-plasmid into a batch bioreactor simulating the human gut, and use it as a spike-in control to assess the performance of Hi-C sequencing and Oxford Nanopore Technologies (ONT)-enabled DNA methylation detection to identify MGE-host pairs. To improve recovery of low-abundance genomes, we used a novel ONT adaptive sampling (AS) strategy that depletes *de novo* assembled, sample-specific high-abundance contigs, rather than relying on reference genomes. This approach led to an approximately twofold enrichment of low-abundance replicons, including the spike-in strain. Methylation-based host assignment failed for the *B. velezensis* MGEs, likely due to the absence of DNA methylation. In contrast, Hi-C successfully linked the phage-plasmid to its host, but not the plasmid, likely due to non-intact cells, and only after removing artefactual signals through bioinformatic processing. For a native *Escherichia coli* strain, Hi-C and methylation data linked it to two plasmids. Selective isolation and whole-genome sequencing of both the native *E. coli* and spike-in *B. velezensis* then confirmed the metagenomic observations. Our results highlight that Hi-C and methylation data can provide powerful insights into MGE-host associations, but their interpretation requires careful computational analysis and biological validation. Moreover, our AS strategy offers a cost-efficient method to boost coverage of low-abundance genomes, improving metagenomic investigation of MGEs in complex microbiomes.

**IMPORTANCE:**

Mobile genetic elements are important contributors to horizontal gene transfer, including of antimicrobial resistance genes. Understanding which microbes carry these mobile elements is vital to assess the spread of resistance. Here, we use a nanopore adaptive sampling approach to increase detection of low-abundance bacteria and mobile elements and use DNA methylation detection and Hi-C sequencing to determine mobile element hosts. By introducing a known bacterium and isolating a native strain, we could evaluate the performance of these methods, indicating that although powerful, they require careful experimental design, interpretation, and validation. However, when combined, these approaches enable a comprehensive investigation of mobile elements and gene transfer dynamics in complex environments.

## INTRODUCTION

Mobile genetic elements (MGEs), including phages and plasmids, play a crucial role in prokaryotic evolution by enabling the rapid spread of genes through horizontal gene transfer (HGT) (1). MGEs frequently carry antimicrobial resistance (AMR) genes and can transmit these to new species in microbial communities (2–6). Therefore, determining their hosts is vital to understanding AMR dynamics and assessing future AMR risks. Traditionally, this is done by cultivation-dependent approaches, such as isolation, to identify the original host, and culture-based mating assays to determine the host range (7). However, as these methods rely on cultivation and the presence of selectable traits such as AMR, their applicability in natural microbial environments is limited, e.g. for uncultivable strains or environments with high background AMR rates, such as the human gut (8). In recent years, several culture-independent tools have been developed that allow studying MGEs in their natural environments (7, 8). Among these, shotgun metagenomic sequencing has emerged as a powerful technique to comprehensively investigate entire microbial communities. However, accurately reconstructing MGEs, their respective hosts, and the broader genetic composition of these communities remains challenging. While long-read metagenomics has enabled significant advances in terms of assembly contiguity and accuracy (9), recovery of low-abundance genomes or MGEs remains difficult at commonly achieved sequencing depths (10, 11). Recently, Oxford Nanopore Technologies (ONT) adaptive sampling (AS) has been applied to alleviate this problem by enriching sequences of interest through the ejection of non-target reads from the nanopore (12–17). In complex natural microbiomes, AS has been applied to increase coverage of low-abundance genomes and MGEs, but these approaches depended on prior availability of external reference sequences (12–14). However, the use of *a priori* available reference genomes results in suboptimal AS performance due to mismatches with the actually present genomes, as demonstrated by Martin et al. (12). Therefore, the target sequences used for AS should be as similar as possible to the actual genomes (12). Once an MGE has been detected, another challenge in metagenomics is defining its host, especially for extrachromosomal MGEs (7, 8). Although computational tools exist to do this based on sequence composition, their performance decreases with increasing taxonomic resolution, especially for broad host range MGEs shared between strains with varying sequence composition (18–21). Recent improvements have enabled long-read sequencing technologies to detect bacterial methylation motifs, caused by methyltransferase (MTase) activity, commonly as part of restriction-modification systems. This information has been applied to attribute MGEs to their hosts by detecting shared methylation motifs (22–25). However, discriminating between different microbiome strains requires MTase activity and sufficient variation in detectable methylation motifs. Hi-C provides another way to predict MGE origins by chemically crosslinking different parts of the genome, capturing their *in vivo* spatial proximity. This has been applied in various environments, including gut microbiomes and wastewater, to comprehensively study MGE host range and transmission (5, 18, 26). However, Hi-C data can be noisy, and many studies do not use spike-in controls or appropriate bioinformatic methods to deal with this noise (27).

In this study, we assessed the performance of the above-described methods in terms of detecting MGEs and determining their host by spiking a well-characterized, niche-atypic *Bacillus velezensis* strain into a batch bioreactor simulating a human gut environment (28, 29). Importantly, the *B. velezensis* strain contains both a linear phage-plasmid and an AMR-carrying plasmid (29). Furthermore, we developed a novel AS approach where *de novo* assembled, sample-specific high-abundance genomes are depleted, thereby favoring the sequencing of low-abundance genomes, including the spike-in. Next, methylation motifs and Hi-C were evaluated for MGE-host linking, using the *B. velezensis* MGEs as an internal control. Finally, we extended the validation of metagenomic observations by selectively isolating both the spiked *B. velezensis* and a native *Escherichia coli* strain, based on the obtained metagenomic information. Isolated data for these strains were then compared to their MAG counterparts. By investigating these methods and providing insights, we aimed to advance the use of metagenomics to study MGEs and their hosts in complex microbiomes.

## RESULTS

### Enhanced recovery of low-abundance genomes and MGEs from metagenomes with adaptive sampling based on sample-specific reference genomes

**Fig 1:**
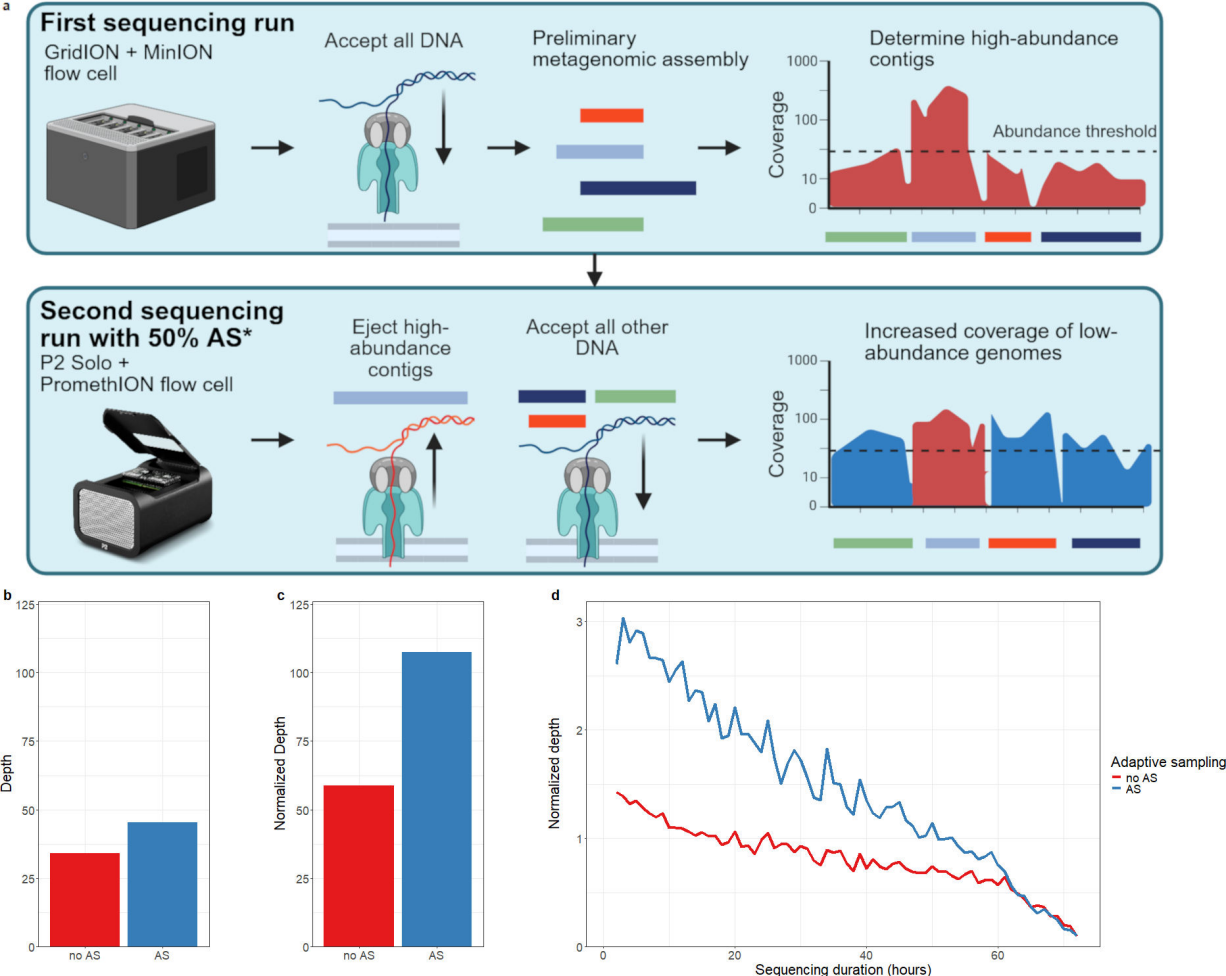
(**a**) Overview of the AS method. A first sequencing run was performed on MinION and followed by metagenomic assembly. Contigs exceeding a certain coverage threshold were retained and depleted with AS in a second sequencing run on a PromethION flow cell. *: Only ~50% of channels were configured to use AS, with the other half running regular sequencing as control. Made on biorender.com, P2 solo image courtesy of Oxford Nanopore Technologies. (**b**) Comparison of spike-in sequencing depth of the *B. velezensis* chromosome with and without the AS method. (**c**) Same as panel b, but normalized for the number of active channels at the start of the experiment. (**d**) Normalized sequencing depth of the spike-in throughout the 72 h experiment, indicating faster decay of throughput in the AS part of the flow cell.

To evaluate metagenomic sequencing for plasmid origin determination in complex microbiomes, we spiked a *B. velezensis* strain, which is not common to the human microbiome environment, carrying high copy numbers of a known plasmid and phage-plasmid into the CoaP batch bioreactor. qPCR detected spike-in plasmid presence after incubation, and re-isolation of the spike-in confirmed the presence of both the plasmid and its host (Table S2). In contrast, and as expected, the control bioreactor was qPCR-negative. Next, we sequenced spiked and control bioreactor DNA extracts on nanopore MinION flow cells with standard conditions, but did not retrieve the *B. velezensis* strain nor its plasmid or phage-plasmid in the spiked sample (Table 1Table 1). To increase sensitivity, we generated a preliminary metagenomic assembly in order to deplete high-abundance contigs (>30× depth) with AS in a next MinION sequencing run (Fig. 1a). While the *B. velezensis* plasmid was detected, horizontal coverage of the chromosome only mildly increased (Table 1). By resequencing on PromethION to increase depth and using AS in ~50% of channels to deplete *de novo* assembled, and hence sample-specific high-abundance contigs, we detected the plasmid and chromosome on both the AS and non-AS part (Table 1). Despite initially containing fewer active channels (1,065 instead of 1,463) and generating a lower yield (40.99 Gb versus 90.69 Gb), the AS part of the flow cell obtained a ~1.3-fold higher coverage depth for *B. velezensis* than the non-AS part (Table 1, Fig. 1b). Normalizing by the initial ratio of active channels increased the enrichment to ~1.8, reflecting the expected enrichment if the AS and non-AS parts had equal active channel counts at the start (Fig. 1c). Interestingly, AS enrichment was higher initially but converged to no enrichment toward the end (Fig. 1d).

**TABLE 1:** Metagenomic detection of spike-in *B. velezensis* and its plasmid and phage-plasmid in both the control and spiked samples using various sequencing methods

Sample	Flow cell	Method	Yield (Gb)	Plasmid coverage breadth (depth)	Phage-plasmid coverage breadth (depth)	Chromosome coverage breadth (depth)
Control	MinION	Rapid	16.6	n.d.^*a*^	n.d.	1.25% (1.84)
Control	MinION	Rapid + AS	3.43	n.d.	n.d.	0.97% (0.07)
Spiked	MinION	Rapid	6.67	35.91% (0.51)	11.77% (0.12)	4.22% (0.22)
Spiked	MinION	Rapid + AS	3.5	67.9% (5.09)	28.87% (0.41)	6.63% (0.15)
Spiked	PromethION: channels 0–1,500	Ligation	90.69	100.00% (1,569.84)	100.00% (319.45)	99.98% (34.44)
Spiked	PromethION: channels 1,500–3,000	Ligation + AS	40.99	100.00% (2,005.15)	100.00% (388.31)	99.98% (43.89)

^*a*^ n.d., not detected.

**Fig 2:**
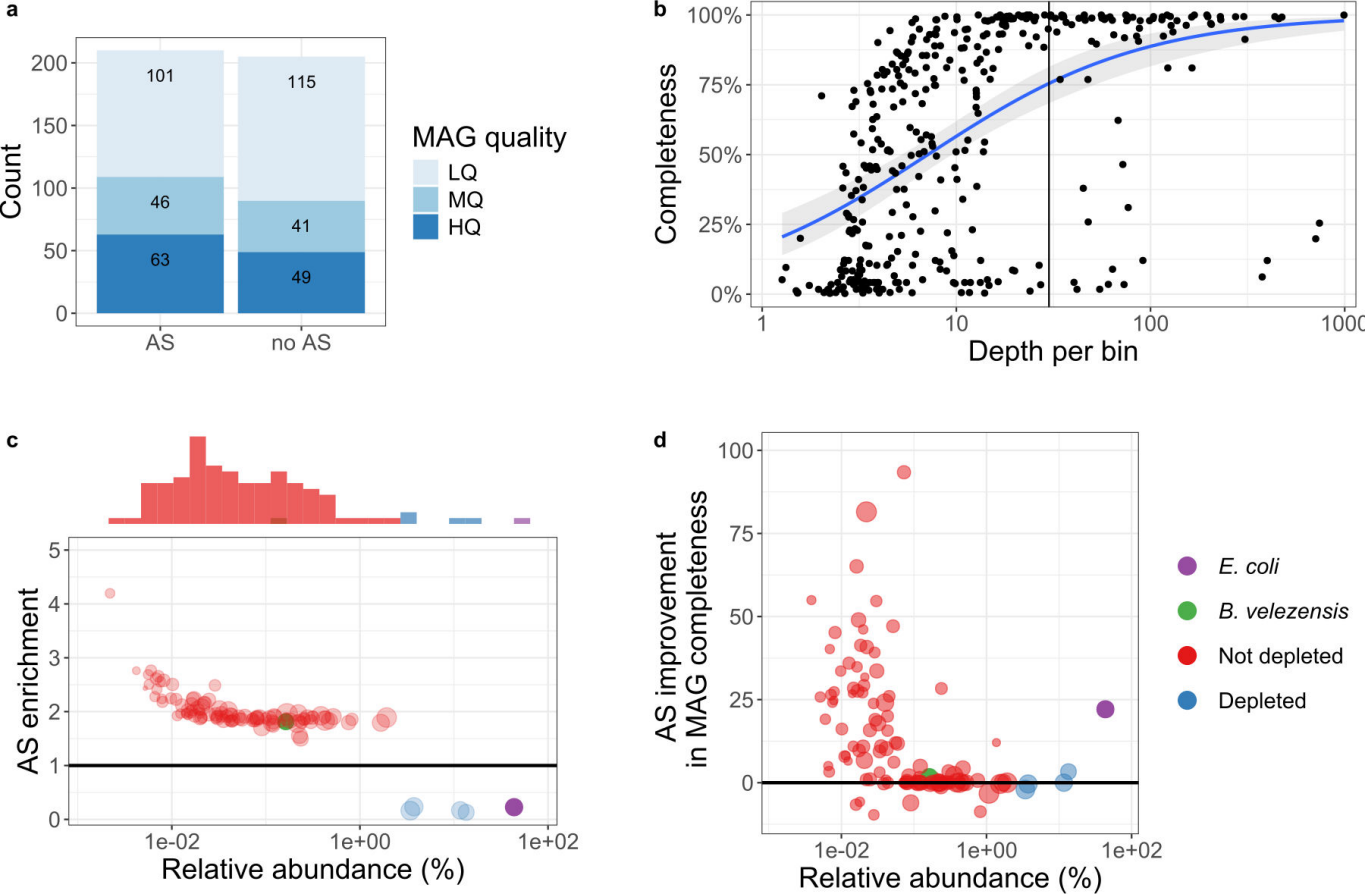
(**a**) Number of MAGs and their quality for the AS and non-AS data. LQ: low quality, <50% complete and <10% contamination; MQ: medium quality, ≥50% complete and <10% contamination; HQ: high quality, >90% complete and <10% contamination. (**b**) MAG completeness by depth for both AS and non-AS assemblies, with a vertical line indicating 30× coverage. The blue line represents a logistic regression, with standard error in gray. (**c**) Normalized enrichment of MAGs (≥50% completeness and <10% contamination) by relative abundance. The histogram on top indicates the distribution of MAGs by relative abundance. (**d**) Comparison of MAG completeness between MAGs from the AS and non-AS metagenomic assemblies.

As our AS method depletes high-abundance contigs, it can enrich other low-abundance genomes alongside the spike-in strain, and potentially improve metagenome assembly. To assess this, we compared the assemblies from the AS and non-AS parts of the PromethION flow cell, only correcting for the initial number of active channels. Despite the lower yield, the AS method recovered 19 additional high- and medium-quality MAGs (≥50% completeness and <10% contamination) compared to the non-AS assembly (Fig. 2a). Next, we assessed the impact of the 30× threshold, beyond which contigs from the preliminary assembly are depleted (Fig. 1a). Between 1× and 30×, completeness strongly increased for the majority of MAGs (Fig. 2b), indicating that 30× is an appropriate threshold in most cases. Still, some MAGs remained incomplete at even higher depths, indicating constraints for assembly and binning beyond depth. We then used non-AS reads to estimate true relative abundances in the higher-quality AS assembly, since AS distorts coverage, and compared AS depth to non-AS depth to calculate enrichment (normalized for initial active pore counts). This revealed that the majority of MAGs were present at low relative abundance (below 1%, Fig. 2c). These low-abundance genomes were a median 2.03-fold enriched (95% bootstrap confidence interval 1.95–2.21). In contrast, high abundance MAGs, such as *E. coli,* were depleted (median 5.95-fold). Also, MAG completeness was significantly higher (one-sided Wilcoxon signed-rank, *P* = 3.303 × 10^−12^) in the AS assembly than in the non-AS assembly, especially for MAGs below 0.1% (Fig. 2d). Finally, we observed a median rejected read length of 675 bases with AS on PromethION, compared to only 445 bases with AS on MinION.

### Methylation-based MGE-origin prediction depends on detectable methylation motifs

**Fig 3:**
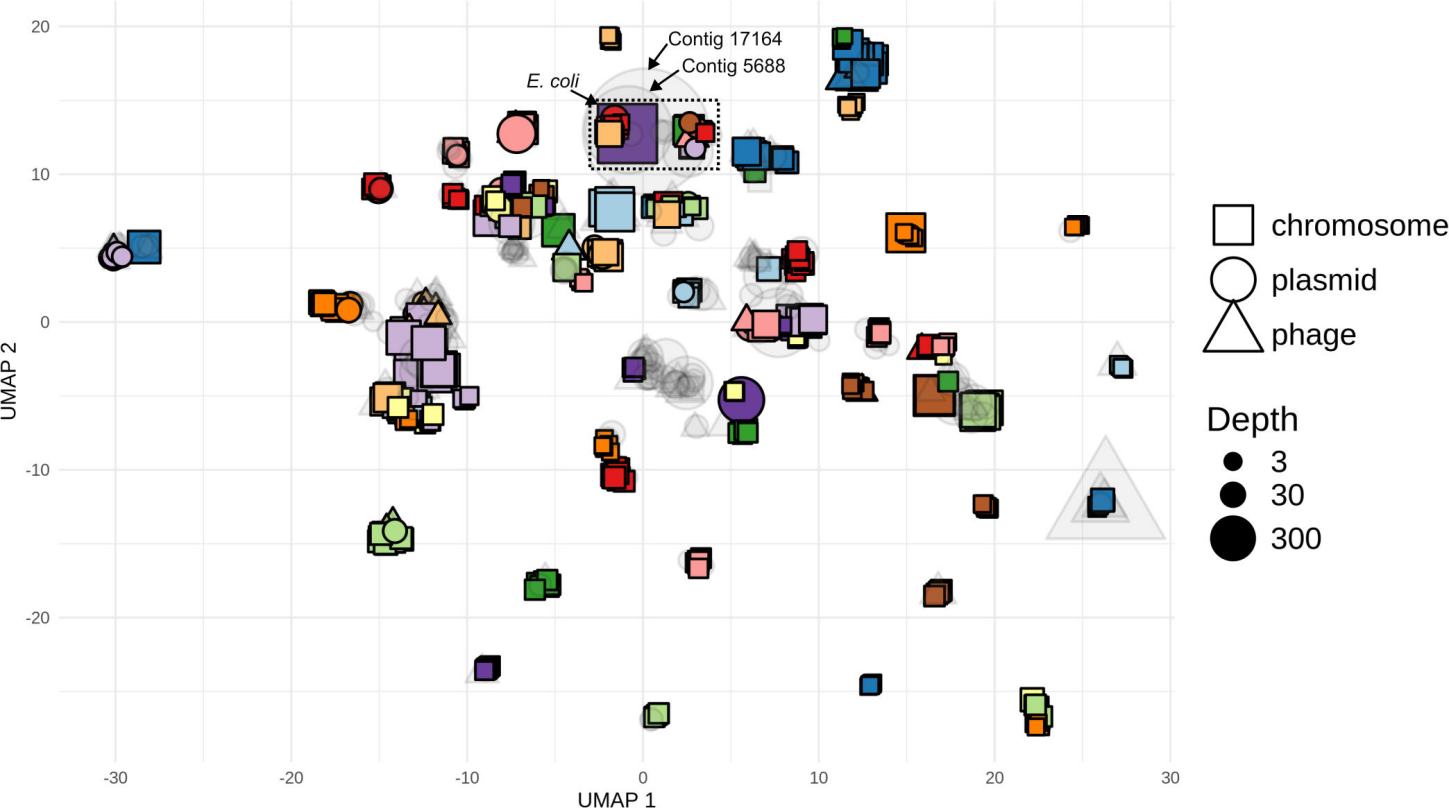
UMAP of methylation patterns in the metagenomic assembly of the PromethION adaptive sampling data set. Mobile elements such as plasmids or phages are indicated by circles and triangles, respectively. Squares represent chromosomal contigs and are colored by MAG. Only MAGs with ≥50% completeness and <10% contamination were considered. Colored symbols indicate binned contigs, while transparent, gray triangles and circles indicate unbinned mobile elements. The *E. coli* MAG (contig 1551, purple square) and two high-depth plasmids (transparent gray circles; contigs 5688 and 17164) are indicated. The dotted square indicates the contigs analyzed further in Fig. 4. *B. velezensis* is absent from this figure, since it contained no identified methylation motifs.

**Fig 4:**
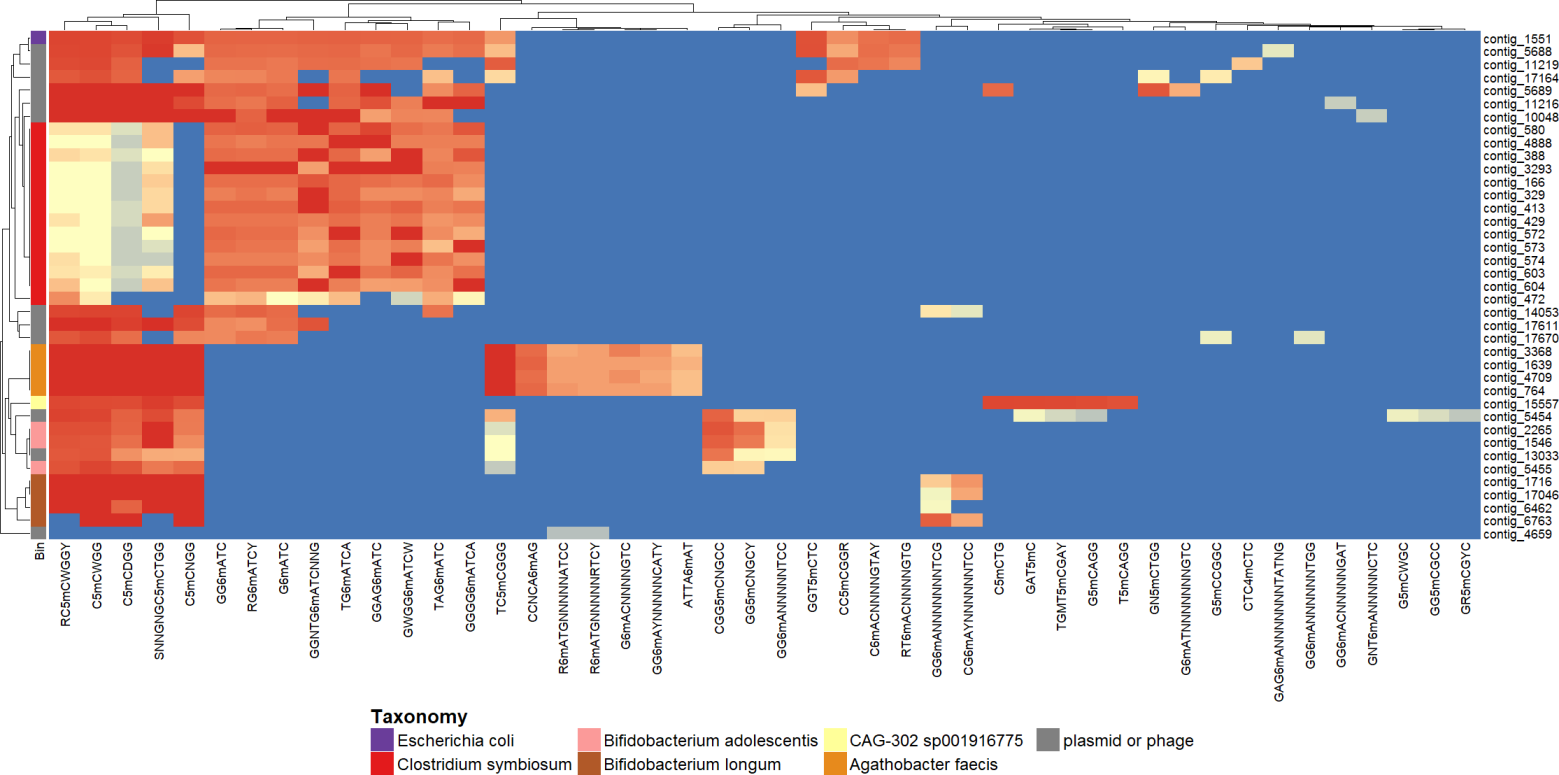
Heatmap of methylation patterns on *E. coli* and contigs from MAGs with similar methylation motifs (dotted square on Fig. 3), clustered by binary distance. Apart from unbinned mobile genetic elements, only MAGs with ≥50% completeness and <10% contamination were plotted.

We mapped both AS and non-AS methylation-called reads to the AS assembly and investigated whether the MGEs carried by the spike-in could be linked to their host through shared methylation motifs. However, in the *B. velezensis* MAG, no methylation motifs were identified, and neither its plasmid nor phage-plasmid could therefore be linked. To ensure sufficient coverage for methylation motif identification, we then performed an ONT sequencing run on the *B. velezensis* isolate. While again no motifs were detected despite the high coverage (4,500×), MicrobeMod (30) detected several potential MTases and type IV restriction enzymes (RE) with high similarity to known REBASE entries (Table 2) (31). In contrast to *B. velezensis*, manual inspection of the *E. coli* MAG and other contigs with similar methylation motifs (Fig. 3) highlighted a potential association between several MGEs (contigs 5688, 17164, 11219, and several others) and a single-contig *E. coli* MAG (contig 1551, Fig. 4). Finally, while methylation sometimes clearly indicated a single host MAG for MGEs, e.g., for *Bifidobacterium adolescentis* and contigs 5454 and 13033 (Fig. 4), the potential origin was more ambiguous for many other MGEs (Fig. 3).

**TABLE 2:** MTases and restriction enzymes detected in the *B. velezensis* spike-in isolate by MicrobeMod^*a*^

Type	(Predicted) motif or activity	HMM e-value	REBASE homolog	REBASE AAI
Type IV RE	cuts m5C	0.0	Ppo12321ORFAP	100.0
Type IV RE	cuts m5C and m6A	1.3e-55	BveP12MrrP	100.0
Type IV RE	cuts m5C	1.5e-12	n.d.	n.d.
Type II MTAse	m5C	7.2e-34	M.BveP22ORF2000P	100.0
Type II MTAse	n.d.	9.4e-52	M.Bve116265ORF11045P	98.0
Type II MTAse	n.d.	5.5e-13	n.d.	n.d.
Type II MTAse	n.d.	9.5e-06	n.d.	n.d.
Type II MTAse	n.d.	9.1e-19	n.d.	n.d.

^*a*^ RE, restriction enzyme; MTase, methyltransferase; HMM, hidden Markov model; REBASE, restriction enzyme database; AAI, amino-acid identity; n.d., not determined.

### Noisy Hi-C data complicates linking spike-in to its MGEs, highlighting the importance of appropriate normalization and filtering

**Fig 5:**
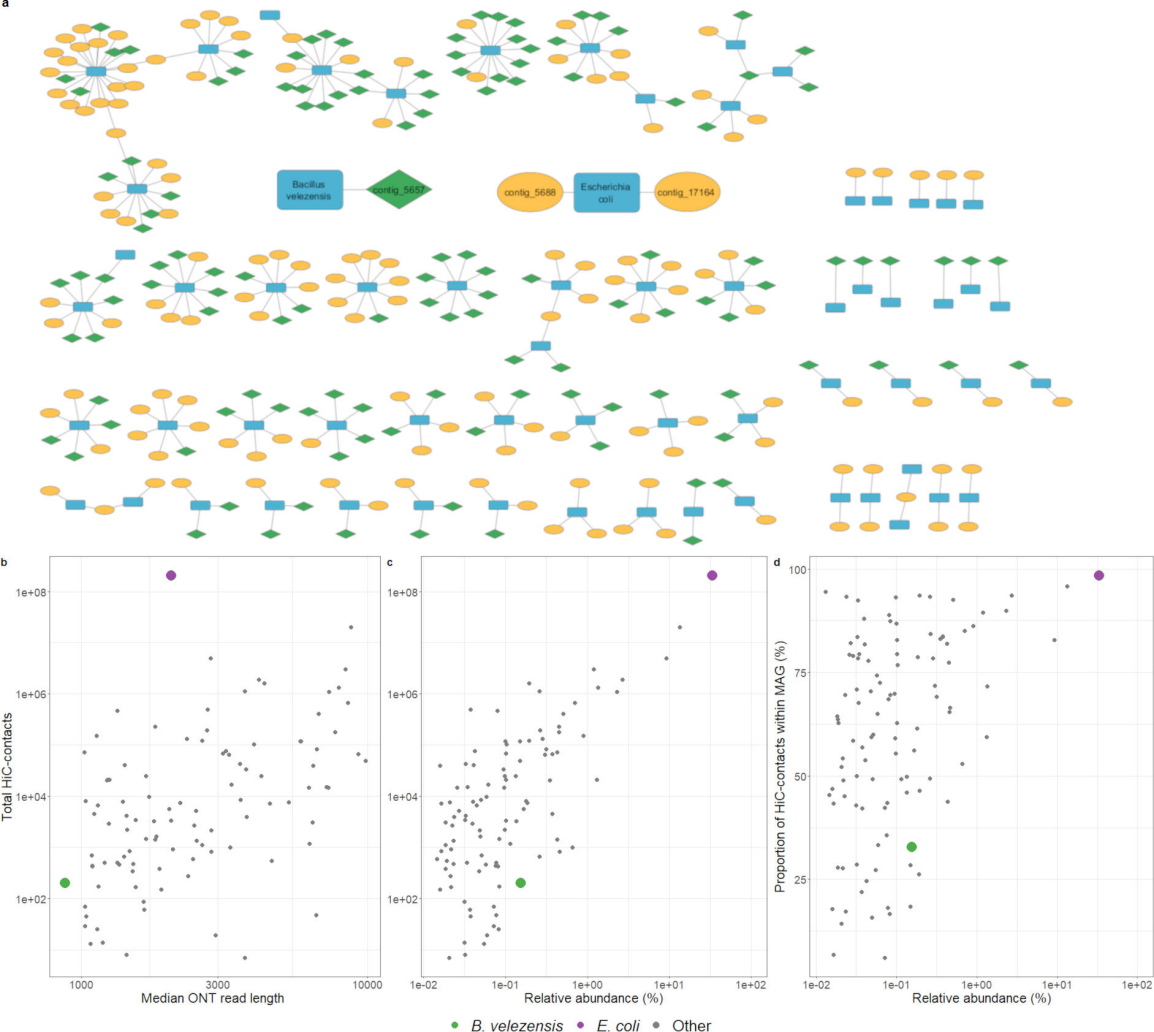
(**a**) Network graph of Hi-C links between plasmids (yellow), phages (green), and their hosts (blue). The *B. velezensis* and *E. coli* MAGs and their associated phages or plasmids are labeled. Mobile elements or MAGs without links (e.g., *B. velezensis* plasmid) are not plotted. (**b**) Number of total raw Hi-C contacts by median ONT read length. (**c**) Number of total raw Hi-C contacts by relative abundance. (**d**) Proportion of intra-MAG Hi-C contacts by relative abundance. In all plots, only high and medium-quality MAGs are included (contamination <10%, completeness >50%).

With DNA methylation detection unable to establish the origin of the plasmid and phage-plasmid carried by the spike-in control, we next sought whether proximity ligation (Hi-C) sequencing could determine this link, using metaCC to correct for various Hi-C biases (32). While no Hi-C links were observed between the *B. velezensis* chromosome and its plasmid, the phage-plasmid could be linked (Fig. 5a). However, this signal could not be discriminated from links to, e.g., *E. coli* in the non-normalized Hi-C data, as only two Hi-C read pairs supported this connection (Fig. S1a). Only after metaCC normalization (32), the correct signal to *B. velezensis* could be distinguished from noise (Fig. S1b). In general, *B. velezensis* had a low number of Hi-C reads compared to other MAGs at comparable relative abundances (0.15%, Fig. 5c), and a majority of these Hi-C contacts mapped to other MAGs (Fig. 5d). Additionally, the low median ONT read length of 874 bases (Fig. 5b) indicates possible DNA degradation (which might be linked to the used growth conditions), as the median length was 2,968 bases in isolate data, and other high and medium-quality MAGs had a median read length of 3,136 bases. In contrast to the *B. velezensis* spike-in, the *E. coli* MAG contained a large number of Hi-C contacts (>10e8) (Fig. 5b and c), most of which were intra-MAG contacts (Fig. 5d). After normalization and filtering, the *E. coli* MAG was linked to two plasmids: contig 5688 and contig 17164 (Fig. 5a), which were previously seen to carry a similar methylation pattern (Fig. 4). Contig 5688 represents a 118 kbp plasmid carrying several AMR genes, including *aadA1* and *blaSHV-1* (Table 3). However, many other contigs with similar methylation patterns (e.g., contig 11219) were not connected to the *E. coli* MAG by Hi-C links. To verify the Hi-C-based links, we selectively cultured the sample on medium supplemented with streptomycin (*aadA1*) and ampicillin (*blaSHV-1*), in order to specifically isolate the strain carrying this plasmid. qPCR was then used to confirm *E. coli* as the species, and the presence of the *aadA1* and *blaSHV-1* genes. The isolate was sequenced separately, which confirmed the presence of the contig 5688 and contig 17164 plasmids, but not the other unbinned MGEs on Fig. 4.

**TABLE 3:** Comparison of *B. velezensis* and *E. coli* isolate assemblies to metagenomic assemblies^*a*^

Isolate contig	Metagenomic detection with geNomad	AMR genes	Reference length (bp)	Metagenomic assembly length (bp)	Number of metagenomic contigs	Reference coverage (%)	Identity (%)
*B. velezensis* chromosome	n.a.^*c*^	*satA*	4,402,877	4,282,440	4	99.71	99.99
*B. velezensis* plasmid	1/7 contigs	*aadA1, bleO*	6,775	7,747	7	71.04	100.00
*B. velezensis* phage-plasmid^*b*^	Yes	/^*d*^	13,899	13,877	1	99.17	100.00
*E. coli* chromosome	n.a.	/	5,112,102	5,112,101	1 (contig 1,551)	100.00	99.99
*E. coli* plasmid 1	Yes	*aadA1, sul1, qacEdelta1, blaSHV-1*	118,065	118,065	1 (contig 5,688)	100.00	100.00
*E. coli* plasmid 2	Yes	/	6,647	13,292	1 (contig 17,164)	100.00	99.99

^*a*^ AMR, antimicrobial resistance**.**

^*b*^ Autocycler did not generate a separate phage-plasmid contig, so we used the contig from the previous reference (accession NZ_OU015426.1).

^*c*^ n.a., not applicable.

^*d*^ /, none detected.

### Validation of metagenomic assembly and metagenomic MGE-host links by isolate whole-genome sequencing

To assess metagenomic detection and assembly of mobile elements and their hosts, we compared the *E. coli* and *B. velezensis* isolate assemblies to their metagenomic counterparts (Table 3). For the spike-in *B. velezensis*, the phage-plasmid was detected in the PromethION data by geNomad and consisted of a single 13.9 kbp contig (5667) covering 99.17% of the reference with 100% identity. For the *B. velezensis* plasmid, seven metagenomic contigs were assembled, covering 71.04% of the reference with 100% identity. Some of these contigs overlapped (Fig. S2a), and only one was reported as a plasmid. The chromosome itself was assembled into four contigs with 99.71% coverage and 99.99% identity to the isolate chromosome. For *E. coli*, two plasmid contigs were assembled in the isolate, and for each, a single metagenomic contig (i.e., 5688 and 17,164) could be aligned with near-perfect coverage and identity to the reference. However, the second plasmid formed a 13.3 kbp contig in the metagenomic assembly, which is a concatenated version of the 6.6 kbp plasmid in the isolate Autocycler assembly (Fig. S2b). We also compared the *E. coli* methylation motifs from the metagenomic data set to those observed in the isolate (see below, Table 4). For motifs with a high methylation degree in the isolate (>80%), Nanomotif found a corresponding MTase, and the same or similar motifs were retrieved from the metagenomic data. In particular, the motif GGT5mCTC discriminated some *E. coli*-MGE pairs from other MAGs in methylation-based clustering (Fig. 4). Its reverse complement, GAG6mACC, was also methylated, but this motif was not detected as such in the MAG. For motifs with <80% methylation in the isolate (e.g., CC5mCGGA), Nanomotif could not identify a MTase.

**TABLE 4:** Comparison of methylation motifs detected in *E. coli* isolate and in its metagenomic-assembled genome

Motif in isolate^*a*^	Degree of methylation (%)	Candidate MTase	Motif in metagenomic assembly^*a*^	Reverse complement
G**6mA**TC	97.9	M.SspPIBDamP	G**6mA**TC (and variations)	
C**5mCW**GG	99.2	M.SflC32DcmP	C**5mCW**GG (and variations)	
GAG**6mA**CC	84.3	M2.Ecos9ORF22615P	n.d.^*b*^	Reverse complement
GGT**5mC**TC	99.0	M2.EcosS3ORF17125P	GGT**5m**CTC (and variations)	
RT**6mA**CNNNNGTG	98.0	M.EcosS3ORF17480P	RT**6mA**CNNNNGTG	Reverse complement
C**6mA**CNNNNGTAY	96.9		C**6mA**CNNNNGTAY	
CC**5mC**GGA	55.1	n.d.	CC**5mC**GGR	Reverse complement
YC**5mC**GGG	64.1	n.d.	TC**5mC**GGG	
CTC**5mC**GGAKRA	73.0	n.d.	n.d.	

^*a*^ Bold indicates the methylated nucleotide.

^*b*^ n.d., not determined.

## DISCUSSION

Understanding HGT dynamics in natural microbial communities is important to assess the genetic exchange of critical traits, such as antimicrobial resistance (7). MGEs play a central role in HGT, but studying them in their natural environments poses several challenges. Many current methods rely on cultivation or can only track a limited number of MGEs or microbes at once (7, 8). In contrast, metagenomics enables a cultivation-independent and comprehensive view of microbiomes and the MGEs within them. However, it depends on sufficient sequencing coverage, which is challenging for low-abundance strains and MGEs (10, 11). Additionally, determining the microbial hosts of MGE is difficult, as DNA extraction disrupts cells, creating a mixed pool of DNA fragments of unknown cellular origins. In this study, we addressed these limitations using three approaches: nanopore AS, detection of shared methylation motifs in MGEs and their host replicons, and Hi-C proximity ligation. Furthermore, we used both a spike-in control and an isolated native strain to validate metagenomic observations.

To address limited sequencing coverage, we used ONT AS to redistribute sequencing capacity from *de novo* sample-specific assembled high-abundant contigs to low-abundant replicons, instead of relying on external, *a priori* known reference genomes for AS. Depleting our d*e novo* assembled contigs resulted in an average of approximately twofold enrichment by yield of low-abundance MAGs, and in particular 1.83-fold for our spike-in control. Other studies report similar enrichments of around twofold by depth but did not normalize by the initial number of active pores (14–17). By enriching all low-abundance genomes, our method led to improved MAG recovery despite reducing overall sequencing yield, similar to results by Sun et al. (14). However, comparing small plasmids in the *B. velezensis* and *E. coli* isolates to their metagenomic counterparts revealed known issues in metagenomic assemblers (33). Also of note, rejected reads were markedly longer for PromethION (675 bases) compared to MinION (445 bases), the latter being in line with other studies (13, 15, 16). These longer rejected reads could indicate bottlenecks in the ability of the GridION to keep up with data generation by the PromethION (run via a P2 Solo device coupled to a GridION). Alleviating these bottlenecks through faster base calling and AS algorithms could reduce decision times, enabling higher enrichment and increasing the benefits of AS (34). Additionally, regular nuclease cleansing and reloading of the flow cell to preserve pore activity (which we did not do) and longer DNA fragments could enable more enrichment (12, 35). Also, our current method requires two flow cells, but could be performed on a single flow cell by pausing the run once sufficient coverage has been reached for microbes at a predetermined abundance threshold. This would be followed by *de novo* assembly and then continuing the run with AS depletion of the high-abundance contigs. Furthermore, dynamic implementations of this method using real-time assembly and real-time updating of the AS decision strategy have also been proposed (12, 13). Nevertheless, the AS method improved assembly of low-abundance genomes and MGEs without requiring higher sequencing depth, thereby increasing cost-efficiency.

While AS can improve recovery of low-abundance MGEs, it does not necessarily resolve the issue of linking extrachromosomal MGEs to their host microbe. To address this, we leveraged ONT-enabled methylation motif detection. This revealed several MGE-*E. coli* pairs sharing common methylated motifs, some exclusive to these pairs, with two MGE*-E. coli* pairs later confirmed by Hi-C and isolation. Most of these shared motifs were further validated by identifying a corresponding MTase and consistency between metagenomic and isolate motifs. Interestingly, CC5mCGGA was one of the *E. coli* motifs for which no MTase was detected and could also be explained by potential erroneous base calling of a C5mCWGG pattern, with a T (W) being base called as 5mC, and the second 5mC as C. In contrast to *E. coli*, the plasmid and linear phage-plasmid of the spike-in control *B. velezensis* could not be linked, as no methylation motifs were reported by the state-of-the-art methylation motif calling tool (36), even in isolate whole-genome sequencing data with ~4,500-fold coverage. Despite the absence of detected motifs, several candidate type II MTases were identified by MicrobeMod. On the other hand, two type IV REs were also detected with high confidence. Since type IV REs are known to cleave at methylated positions with poor sequence specificity (37), we suspect that these would be incompatible with active DNA methylation, potentially explaining why our *B. velezensis* strain seems to lack any type II methylation motifs. In this case, methylation-based MGE host linking would indeed not be feasible. For many other MGEs, methylation-based host attribution was ambiguous. These observations illustrate that methylation-based MGE-host attribution requires the presence and activity of methyltransferases, along with sufficient variation in detectable methylation motifs, which is not always the case (23, 38).

Whereas the resolution of DNA methylation binning is inherently limited by the number and types of methylation motifs, Hi-C sequencing enables a higher resolution by directly preserving DNA proximity information before lysis and subsequent DNA extraction. Nonetheless, Hi-C data needs to be interpreted with caution, as McCallum et al. demonstrated frequent artefactual Hi-C links and highlighted the importance of spike-in controls (27, 39). In our case, we used a thoroughly characterized *B. velezensis* spike-in control and could link it to its phage-plasmid, which was not possible with methylation data. However, no links were found to its plasmid, and the link to the phage-plasmid was supported by only two Hi-C contacts. Furthermore, before data processing, most raw spike-in Hi-C read pairs originating from the chromosome or the phage-plasmid connected them to other MAGs, indicating likely artefactual links. One possible explanation is that most *B. velezensis* DNA was present extracellularly, resulting in fewer and more random Hi-C contacts, as extracellular DNA is more likely to come into physical proximity with DNA from other strains (27, 40). Extracellular DNA degradation could also explain the shorter *B. velezensis* ONT read lengths compared to other species (41, 42). More stringent washing steps or DNase treatment could improve the Hi-C signal-to-noise ratio by removing extracellular DNA before the crosslinking step. After normalization and filtering of the raw Hi-C data, the artefactual Hi-C links to the spike-in were removed, highlighting the power of noise reduction and normalization strategies (27, 32). For *E. coli,* the processed Hi-C results were clearer and indicated that it hosted two plasmids, which were also seen in methylation-based linking, with one of these carrying several AMR genes. Using this information, we selectively isolated streptomycin- and ampicillin-resistant *E. coli* and then confirmed plasmid presence through isolate sequencing. This serves as proof of concept of how methylation motif detection and Hi-C can be applied in complex communities to obtain information on MGE hosts and to subsequently isolate MGEs of interest. Both methods have advantages and disadvantages, with Hi-C having higher resolution but depending on cellular integrity and accurate noise removal, while methylation detection is less dependent on cellular integrity, but on active MTases and sufficient variation in detectable methylation motifs.

While our study was performed on a single bioreactor sample, it reflected natural community structures, as it originated from a human donor and had at least 60 different genomes and a highly uneven distribution. As uneven distributions are common in microbial ecology (43), our AS approach could improve recovery of rare genomes and MGEs across different environments. Nevertheless, methylation binning efficiency likely depends on community MTase diversity, and Hi-C sequencing might require additional sample collection and processing steps in more complex sample matrices, e.g., to remove noise originating from extracellular debris.

In conclusion, our results demonstrate how combining nanopore AS, methylation profiling, and Hi-C sequencing could enable recovery of MGE-host pairs at low abundances. By including a spike-in control and isolating a native microbiome member, we showed that these approaches can generate biological insights but require careful experimental design and validation. Future work using ONT metagenomic sequencing could benefit from increased read lengths, faster AS algorithms, and improved DNA modification calling. Hi-C sequencing results could be improved by separating cellular debris from truly intact cells and using appropriate bioinformatic methods for interpretation. Taken together, these advancements will facilitate accurate, comprehensive, and cost-efficient tracking of MGEs in natural microbiomes, which is crucial to monitor the spread of AMR.

## MATERIALS AND METHODS

### *In vitro* microbiome cultivation and spiking

The human fecal sample was collected by ProDigest (Ghent, Belgium) from an anonymized individual according to the ethical approval of the University Hospital Ghent. It was then stored anaerobically at 4°C until processing (within 48 h after sample collection). The fecal suspension was sparged with nitrogen gas before flash-freezing at −80°C, using a modified version of the cryoprotectant used by Hoefman et al. (44). One milliliter of a 7.5% suspension of the fecal suspension was inoculated into the Colon-on-a-Plate (CoaP, Prodigest) reactor, in 10 mL total volume. The *B. velezensis* strain, described in reference 29, was grown overnight in Brain-Heart infusion broth (BHI, ThermoFisher Scientific) at 37°C, with 5 µg/mL of kanamycin (Sigma-Aldrich). One hundred microliters were then transferred to a 250 mL flask containing 25 mL BHI (ThermoFisher Scientific) and grown to OD_600_ 0.2, corresponding to 10^8^ CFU/mL. One milliliter was centrifuged at 5,000 × *g* for 1 min, washed with 1 mL of PBS (ThermoFisher Scientific), and pelleted at 5,000 × *g*. The pellet was resuspended in 100 µL PBS and added to the CoaP reactor. Another reactor was not spiked (negative control). Both reactors were then incubated for 48 h at 37°C under anaerobic conditions and mild shaking (90 rpm).

### Sampling and DNA extraction

For Hi-C sequencing and isolation, 500 µL of CoaP fluid was combined with 500 µL of the cryoprotectant described above, and flash-frozen at −80°C. For DNA extraction, 1 mL of CoaP fluid was pelleted (at 5,000 × *g*) and resuspended in 250 µL of DNA/RNA shield (Zymo Research). DNA was extracted using a modified version of the three-peaks extraction method (45), as described in Bloemen et al. (25). For DNA extraction from *B. velezensis* and *E. coli* isolates, colonies were inoculated in 10 mL Luria-Bertani (LB) broth (Sigma-Aldrich) with appropriate antibiotics (5 µg/mL kanamycin and 10 µg/mL streptomycin, respectively), and incubated overnight at 37°C with shaking. Next, 1 mL was pelleted at 5,000 × *g*, resuspended in 250 µL of DNA/RNA shield (Zymo Research), and DNA was extracted according to the same method as for the CoaP sample.

### qPCR and isolation of spiked *B. velezensis* strain and native *E. coli* strain

qPCR to detect *B. velezensis* was done by adding 5 µL of extracted DNA to 20 µL qPCR mastermix containing 1× Ssoadvanced universal probes mastermix (Biorad) and the Bvel_plasmid primer/probe set (28) (Table S1). A Cq >35 was interpreted as negative (46). *B. velezensis* was re-isolated by inoculating 10 µL of cryopreserved CoaP fluid in 10 mL LB with 5 µg/mL kanamycin, and growing overnight at 37°C while shaking, followed by three overnight passages in 5 µg/mL kanamycin LB. Dilution series were then plated on 5 µg/mL kanamycin LB agar. Colonies were picked and incubated in 100 µL nuclease-free water (Invitrogen) for 10 min at 95°C. Five microliters of this served as a template for qPCR, containing 20 µL of 1× SYBR Green Real Time PCR Mastermix (Biosense) and the Bvel plasmid and chromosome primers (Table S1). For *E. coli* isolation, 10 µL of cryopreserved CoaP sample was inoculated in 10 mL LB with 10 µg/mL streptomycin, grown overnight at 37°C with shaking. Dilutions were plated on MacConkey agar (Oxoid) with streptomycin (10 µg/mL) and ampicillin (35 µg/mL), and colonies were screened for *blaSHV, sul1,* and *uidA* using primers as outlined in Table S1, using the same method as for *B. velezensis* colony qPCR. For all assays, the PCR protocol was 10 min at 95°C, followed by 45 cycles of 15 s at 95°C and 1 min extension at 60°C.

### Nanopore sequencing, adaptive sampling, and base calling

Metagenomic libraries were generated according to the manufacturer’s instructions using ONT rapid sequencing (SQK-RAD114) for MinION R10.4.1 flow cells (FLO-MIN114), and ONT ligation sequencing (SQK-LSK114) for PromethION (FLO-PRO114M). Samples were sequenced on a GridION or P2Solo connected to GridION (ONT) for 72 h. Isolates were sequenced with SQK-LSK114 on MinION R10.4.1 flow cells. Dorado 0.9.1 and the dna_r10.4.1_e8.2_400bps_sup@v5.0.0 model were used for base calling, with option “--modified-bases 4mC_5mC 6mA,” and methylation models v5.0.0_4mC_5mC@v3 and v5.0.0_6mA@v3. Reads were filtered for Q >10 and length >500 bp using seqkit v2.3.1 (47). The AS runs on metagenomic samples were carried out as follows (Fig. 1): reads were assembled from MinION data sets without AS using Flye v2.9.4 (48), with options “--nano-hq --meta -i 1 --read-error 0.03.” Contigs with coverage >30× were used for the adaptive sampling deplete feature in MinKNOW (35). In the MinION AS runs on metagenomic libraries, all channels were used for AS, while on PromethION, channels 1–1,500 were selected for AS, with the remaining channels serving as control. To normalize coverage depths, we used the number of active channels at the start, determined from “pore_activity.csv.”

### Detection of *B. velezensis* spike-in

Reads were mapped to the *B. velezensis* reference genome (ncbi accession GCF_907164735.1) with Minimap2 v2.28 (49), and filtered with SAMtools v1.21 (50), with flags “-F0x904.” Finally, a filtering step using pysam v0.22.1 removed reads with <90% identity or <80% mapped bases.

### Proximity ligation (Hi-C) sequencing and read filtering

Proximity ligation was carried out using Proximeta (Phase Genomics), following the Proximeta v4 protocol. The resulting libraries were sequenced on a P2 flow cell on NextSeq 1000, generating 424 M 150 bp paired-end reads. For Hi-C reads, adaptor sequences, low-quality reads, and PCR duplicates were removed using the bbduk, clumpify, and repair scripts from BBmap v38.44 (51), according to the methods by Du and Sun (32). Hi-C reads were then mapped to the ISFinder database, downloaded from https://github.com/thanhleviet/ISfinder-sequences (52) using BWA-MEM v0.7.17 (53) with options “E50 -L0,” and reads with ≥60% coverage and ≥99% identity were removed to remove artefactual Hi-C links (27).

### Metagenomic analysis

The PromethION data were split according to the AS and non-AS parts of the flow cell. Next, Flye (48) was used with options “--nano-hq --meta -i 2 --read-error 0.03.” Contigs were polished using Medaka v2.0.0 with the dna_r10.4.1_e8.2_400bps_sup@v5.0.0 model. Hi-C reads were mapped to the assembly using BWA-MEM v0.7.17, with preset “−5SP.” Minimap2 v2.28 (49) was used with parameters “-ax map-ont -y” to map ONT reads back to the assembly. Binning was done with MetaBAT2 v2.15 (54), GraphMB v0.1.5 (55) with parameters “--mincontig 3000 --assembly_type flye --vamb --minbin 250000” and SemiBin v2.1.0 (56) with parameters “--environment global --sequencing-type long_read --compression=none.” MetaCC v1.2.0 was used for Hi-C binning, with options “-e MluCI -e Sau3AI” for the normalization step (32). DASTool v1.1.6 (57) with parameter “--score_threshold 0” was used to select the best binning results. Methylation pileups were generated using Modkit v0.3.0, followed by Nanomotif v0.6.2 (24) “motif_discovery” with parameters “--read-level-methylation --threshold-valid-coverage 1,” and “include_contig” with “--run_detect_contamination.” MAGs were classified using “gtdbtk classify_wf” from GTDB-Tk v2.3.2 with GTDB database release 214 (58). MAG quality was assessed with “checkm lineage_wf” from CheckM v1.2.2 (59). MAGs were matched across different metagenomic assemblies by taking the closest FastANI hit with size >500 kbp (60), and compared to isolate assemblies using Mummer v4.0.0rc1 (61). R v4.4.3 and ggplot2 were used to visualize assembly statistics.

Plasmid and phage elements were identified with geNomad v1.8.0 (62). Prophage contigs with <50% of prophage content were excluded from MGE-host contact maps, as these are more host than prophage. To generate MGE-host Hi-C contact graphs, MetaCC-normalized data were used, and the number of normalized contacts was aggregated by MAG. Contigs from bins with <50% completeness or >10% contamination were considered “unbinned.” Spurious Hi-C contacts were removed by filtering out MGE-bin links with <300,000 normalized contacts, and only retaining links representing ≥30% of all contacts to a given MGE. The final graph was visualized in Cytoscape v3.10.2 (63). For methylation analysis, the same thresholds were used (<50% completeness or >10% contamination) for regarding contigs as unbinned. Only motifs detected at the bin level were retained, and motifs with <25% methylation in a bin (mean of median methylation degree per contig, weighted by contig length) or contig were removed. R v4.4.3 and the R packages ComplexHeatmap v2.10.0 (64) and UMAP v0.2.10.0 (65) were then used to visualize methylation data. For UMAP, a cosine distance matrix was used, while the Jaccard binary distance was used for the heatmap clustering.

### Isolate assembly and methylation analysis

For both the *B. velezensis* spike-in and the native *E. coli*, we generated long-read assemblies using ONT R10.4.1 data (obtained as described above) with Autocycler v0.3.0 (66), using the wrapper script https://github.com/rrwick/Autocycler/blob/main/pipelines/autocycler_wrapper_by_iskold/autocycler_wrapper.sh with default settings for *E. coli*. A modified version was used to obtain an assembly for *B. velezensis*. Flye v2.9.4 was used as described above with all reads. For the other Autocycler assemblers, reads were downsampled to 10% (~450× coverage) for efficiency, and a single assembly was made with each assembler. For both *E. coli* and *B. velezensis*, all reads were used for methylation calling and motif detection with Nanomotif as described above. For *B. velezensis,* MicrobeMod v1.1.0 was additionally used to detect MTase genes, since no methylation motifs were identified.

## Data Availability

The sequencing data generated in this study have been deposited in ENA under project PRJEB95107. The bioinformatic pipeline for metagenomic assembly and Hi-C data processing can be found at https://github.com/brambloemen/ONT_MAG_pipeline/tree/MGE-host_linking_study.

## SUPPLEMENTAL MATERIAL

Supplemental tables and figures msystems.01282-25-s0001.docx Table S1 and S2; Figures S1 and S2.

ASM does not own the copyrights to Supplemental Material that may be linked to, or accessed through, an article. The authors have granted ASM a non-exclusive, world-wide license to publish the Supplemental Material files. Please contact the corresponding author directly for reuse.
