# Supplementary material for: Recovery and microbial host assignment of mobile genetic elements in complex microbiomes: insights from a spiked gut sample
Source: mSystems. 2026 Jan 26;11(2):e01282-25. doi: 10.1128/msystems.01282-25 (PMC12911394; doi:10.1128/msystems.01282-25)
Supplement: Supplemental tables and figures — Table S1 and S2; Figures S1 and S2. [file msystems.01282-25-s0001.docx]

| **Target** | **Name** | **Sequence** | **Concentration** | **Annealing temperature** | **Reference** |
| --- | --- | --- | --- | --- | --- |
| *B. velezensis* plasmid | Bvel_plasmid_F | CGAGAATGCAGCTGAAACAG | 400 nM | 60°C | (1, 2) |
|  | Bvel_plasmid _P | FAM-GGACGGACAGATCAAGAACTGTTATGG- TAMRA | 200 nM |  |  |
|  | Bvel_plasmid _R | CATATGCTCGGGGAATTTATCT | 400 nM |  |  |
| *B. velezensis* chromosome | Bvel_F | GGTTGGAACCTACGGGTACT | 400 nM | 60°C | This study |
|  | Bvel_R | ATTCCGGTCTCACTGTTTACG | 400 nM |  |  |
| *E. coli* plasmid *blaSHV* | *blaSHV*_F | AGCCGCTTGAGCAAATTAAACT | 400 nM | 60°C | (3) |
|  | *blaSHV*_R | CATCATGGGAAAGCGTTCATC | 400 nM |  |  |
| *E. coli* plasmid  *sul1* | *sul1*_F | CGCACCGGAAACATCGCTGCAC | 400 nM | 60°C | (4) |
|  | *sul1*_R | TGAAGTTCCGCCGCAAGGCTCG | 400 nM |  |  |
| *E. coli uidA* (confirmation of *E. coli*) | *uidA3*_F | GCAGTTTCATCAATCACCAC | 250 nM | 60°C | (5) |
|  | *uidA3*_R | CTCCTACCGTACCTCGCATTAC | 250 nM |  |  |

**Table S1:** overview of qPCR assays

**Table S2:** qPCR results. n.d.: not detected

| **Target** | **Sample** | **Mean Cq of triplicate (±standard error)** | **Positive tests (Cq <35)** | **Number of tests with Cq < 45** |
| --- | --- | --- | --- | --- |
| *B. velezensis* plasmid | Control | 38.33 | 0/3 | 1/3 (2/3 n.d.) |
| *B. velezensis* plasmid | Spiked | 22.77 ± 0.22 | 3/3 | 3/3 |


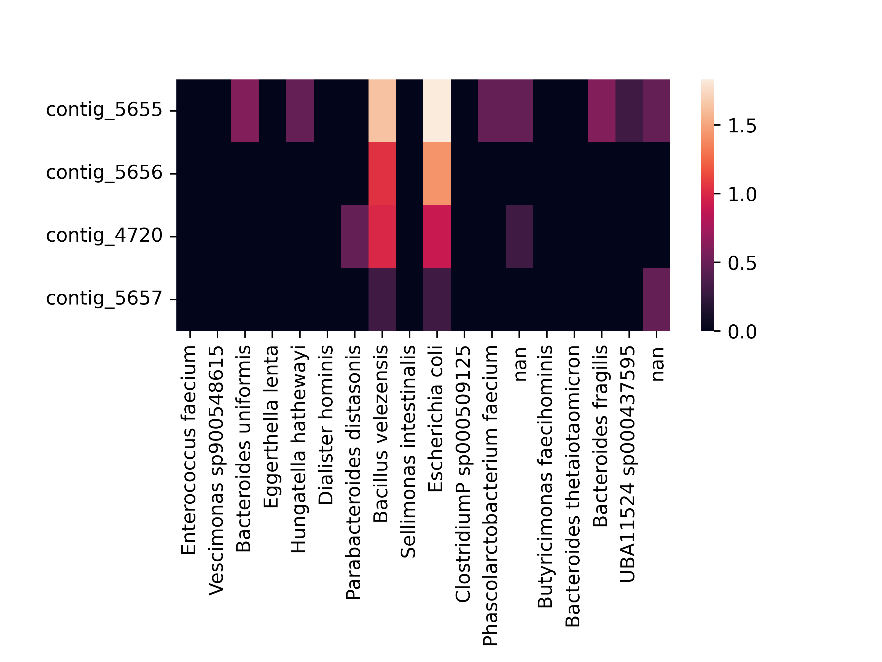


**a**


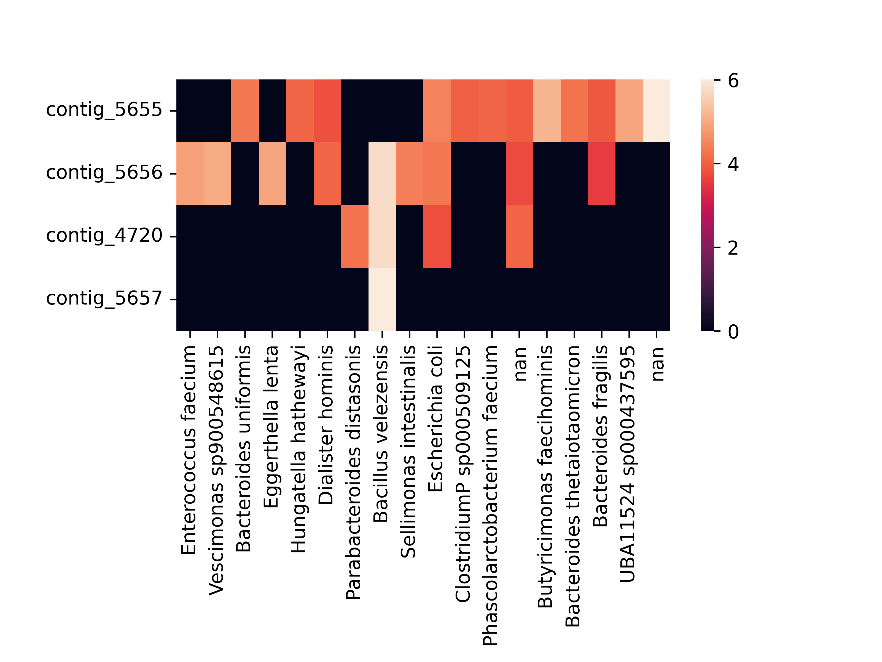


**b**

**Figure S1:** Heatmap of **a)** raw Hi-C contacts (log₁₀-transformed) and **b)** metaCC-normalized Hi-C contacts (log₁₀-transformed) between the *B. velezensis* metagenome-assembled genome (MAG) (contigs 5655, 5656, 4720), its extrachromosomal phage (contig 5657), and various other MAGs. nan: unclassified MAGs


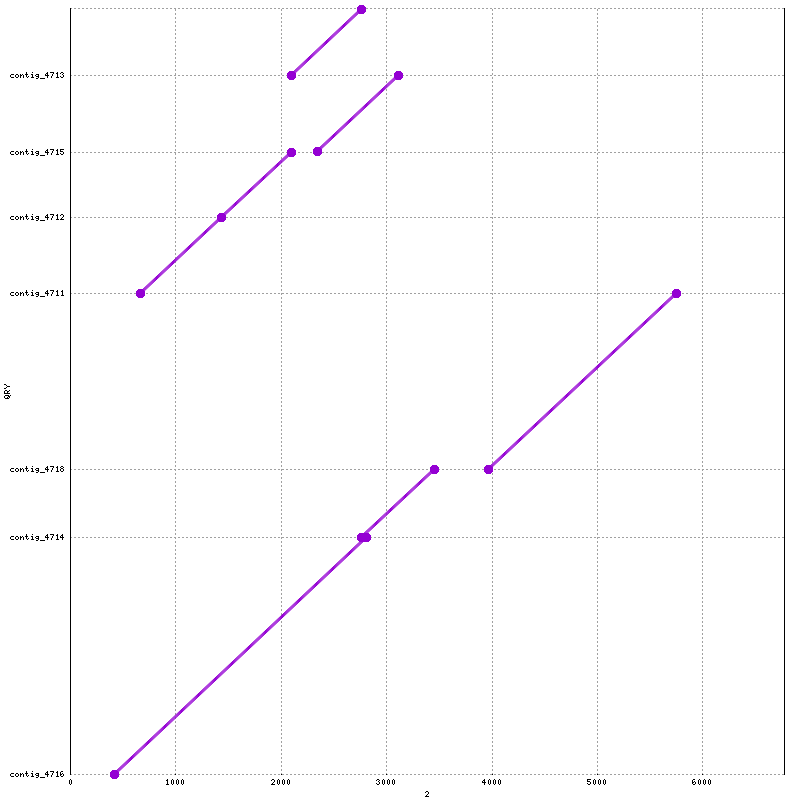

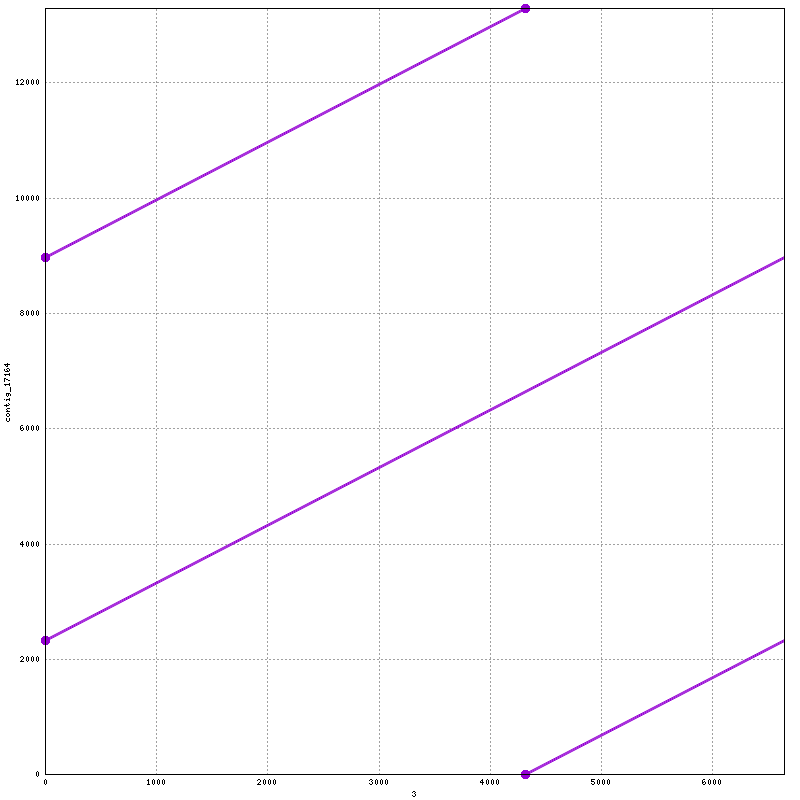


**b**

**a**

**Figure S2:** Mummerplot comparison of metagenomic assembly of **a)** *B. velezensis* plasmid and **b)** *E. coli* plasmid to their autocycler counterparts.

**References**

1. D’aes J, Fraiture M-A, Bogaerts B, De Keersmaecker SCJ, Roosens NHC, Vanneste K. 2021. Characterization of Genetically Modified Microorganisms Using Short- and Long-Read Whole-Genome Sequencing Reveals Contaminations of Related Origin in Multiple Commercial Food Enzyme Products. 11. Foods 10:2637.

2. Fraiture M-A, Bogaerts B, Winand R, Deckers M, Papazova N, Vanneste K, De Keersmaecker SCJ, Roosens NHC. 2020. Identification of an unauthorized genetically modified bacteria in food enzyme through whole-genome sequencing. Sci Rep 10:7094.

3. Hammond DS, Schooneveldt JM, Nimmo GR, Huygens F, Giffard PM. 2005. blaSHV Genes in Klebsiella pneumoniae: Different Allele Distributions Are Associated with Different Promoters within Individual Isolates. Antimicrob Agents Chemother 49:256–263.

4. Tran TQ, Park M, Lee JE, Kim SH, Jeong J-H, Choy HE. 2023. Analysis of antibiotic resistance gene cassettes in a newly identified Salmonella enterica serovar Gallinarum strain in Korea. Mobile DNA 14:4.

5. Barbau-Piednoir E, Denayer S, Botteldoorn N, Dierick K, De Keersmaecker SCJ, Roosens NH. 2018. Detection and discrimination of five E. coli pathotypes using a combinatory SYBR® Green qPCR screening system. Appl Microbiol Biotechnol 102:3267–3285.
